# Favorable effect of renal denervation on elevated renal vascular resistance in patients with resistant hypertension and type 2 diabetes mellitus

**DOI:** 10.3389/fcvm.2022.1010546

**Published:** 2022-12-14

**Authors:** Musheg Manukyan, Alla Falkovskaya, Victor Mordovin, Stanislav Pekarskiy, Irina Zyubanova, Ekaterina Solonskaya, Tamara Ryabova, Simzhit Khunkhinova, Anastasia Vtorushina, Sergey Popov

**Affiliations:** Tomsk National Research Medical Center, Russian Academy of Sciences, Cardiology Research Institute, Tomsk, Russia

**Keywords:** renal denervation, renal function, renal hemodynamics, renal resistive index, type 2 diabetes mellitus, resistant hypertension

## Abstract

**Objective:**

To assess the effect of renal denervation (RDN) on renal vascular resistance and renal function in patients with drug-resistant hypertension (HTN) and type 2 diabetes mellitus (T2DM).

**Materials and methods:**

Fifty-nine patients (mean age 60.3 ± 7.9 years, 25 men) with resistant HTN [mean 24-h ambulatory blood pressure (BP) 158.0 ± 16.3/82.5 ± 12.7 mmHg, systolic/diastolic] and T2DM (mean HbA1c 7.5 ± 1.5%) were included in the single-arm prospective study and underwent RDN. Renal resistive index (RRI) derived from ultrasound Doppler; estimated glomerular filtration rate (Chronic Kidney Disease Epidemiology Collaboration formula), office and 24-h ambulatory BP were measured at baseline, 6, and 12 months after RDN to evaluate the respective changes in renal vascular resistance, renal function, and BP during treatment.

**Results:**

Forty-three patients completed 12 months follow-up. The RRI changed depending on the baseline value. Specifically, the RRI decreased significantly in patients with elevated baseline RRI values ≥ 0.7 {*n* = 23; −0.024 [95% confidence interval (CI): −0.046, −0.002], *p* = 0.035} and did not change in those with baseline RRI < 0.7 [*n* = 36; 0.024 (95% CI: −0.002, 0.050), *p* = 0.069]. No significant change was observed in eGFR whereas BP was significantly reduced at 12 months after RDN by −10.9 (95% CI: −16.7, −5.0)/−5.5 (95% CI: −8.7, −2.4) mmHg, systolic/diastolic. No relationship was found between the changes in RRI and BP.

**Conclusion:**

Our study shows that RDN can decrease elevated renal vascular resistance (RRI > 0.7) and stabilize kidney function in patients with RHTN and T2DM independently of its BP-lowering effect.

## Introduction

Type 2 diabetes mellitus (T2DM) and hypertension (HTN) are major contributors to the development and progression of chronic kidney disease (CKD) (1). Impairment of renal function greatly increases the incidence of renal and cardiovascular complications (2). Uncontrolled HTN in patients with T2DM accelerates kidney damage (3). Elevated renal resistive index (RRI) is a marker of subclinical renal damage and reflects an increase in renal vascular resistance (4). Furthermore, an RRI value > 0.7 is closely associated with poor renal outcomes in patients with HTN (5–7), CKD (8), and T2DM (9, 10). In addition, increased RRI in patients with HTN correlates with atherosclerotic factors (11), target organ damage (12), and cardiovascular outcomes in HTN (7), including true resistant HTN (RHTN) (13). Recent studies have shown that renal denervation (RDN) may reduce RRI, improve intrarenal blood flow, and stabilize renal function in patients with RHTN (14, 15). However, these effects may be even more important for patients with a combination of RHTN and T2DM, who have the highest risk of renal and cardiovascular complications (16–19). This study aimed to test whether RDN improves renal vascular resistance and has a favorable effect on renal function in patients with a combination of RHTN and T2DM.

## Materials and methods

**FIGURE 1:**
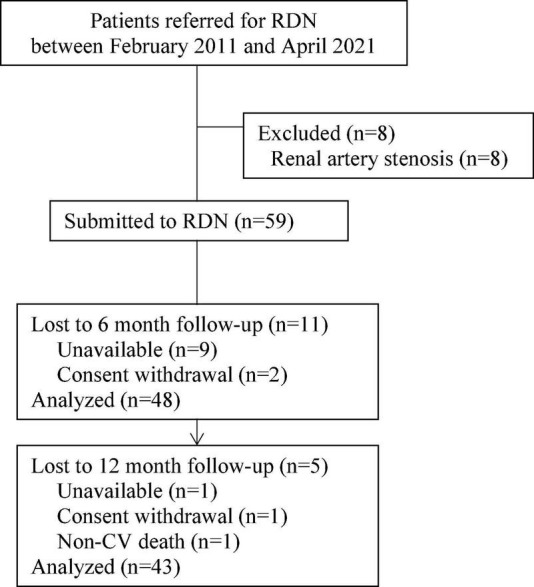
Study flow chart. From the total number of patients (*n* = 67), 59 patients underwent renal denervation (RDN), after the exclusion of eight patients due to renal artery stenosis.

Fifty-nine patients with RHTN and T2DM were included in a single-center prospective interventional study and underwent RDN at the Research Institute of Cardiology, Tomsk National Research Medical Center, between February 2011 and April 2021. The study was performed in accordance with applicable national and international standards (Good Clinical Practice and the principles of the Declaration of Helsinki). The study protocol was approved by the local ethics committee. All patients provided written informed consent prior to inclusion in the study. Eligible patients included men and women 18–80 years of age with office systolic blood pressure (SBP) ≥ 140 mmHg, while receiving three or more antihypertensive medications without changes for 3 months prior to enrollment. RHTN was verified according to European Society of Cardiology and European Society of Hypertension (ESC/ESH) Guidelines for the management of HTN (20). Diagnosis of T2DM was confirmed as recommended by ESC Guidelines on diabetes, pre-diabetes, and cardiovascular diseases developed in collaboration with the European Association for the Study of Diabetes (21). The exclusion criteria were pseudo-resistant HTN, secondary HTN, 24-h ambulatory SBP less than 135 mmHg, severe T2DM, glycosylated hemoglobin (HbA1c) > 10%, estimated glomerular filtration rate (eGFR) < 30 mL/min/1.73 m^2^, pregnancy, renal artery anatomy ineligible for treatment, and severe comorbidity significantly increasing the risk of the intervention according to investigator’s assessment. Information about drug therapy was obtained through an interview. The study flow chart is present in Figure 1.

### Assessment of renal vascular resistance

The RRI was calculated as the relative pulsatile difference in linear flow velocities (peak systolic velocity–end-diastolic velocity)/peak systolic velocity measured by renal triplex ultrasound at the level of the segmental arteries, according to the standard technique (4). The average value of the RRI from the upper, middle, and lower kidney segments was used. The RRI values for the left and right kidneys were averaged, office and 24-h ambulatory BP were measured according to standard guidelines (20). Isolated systolic HTN (ISHTN) was defined as an elevated office systolic BP (> 140 mmHg) with a normal diastolic BP (< 90 mmHg). Renal function was assessed using the eGFR (CKD-EPI formula). CKD was defined as eGFR < 60 mL/min/1.73 m^2^. According to the study protocol, RRI, renal function, office and 24-h ambulatory BP were measured at baseline and at 6 and 12 months post-procedure. The patients were instructed not to change their medication regimen for the duration of the study.

### Outcomes

The primary outcome was reduction in RRI for segmental renal arteries from baseline to 12 months post-procedure, and the secondary outcomes were changes in eGFR, and office and ambulatory BPs from baseline to 6 and 12 months after RDN. Improvement in renal vascular resistance was categorized as a decrease in RRI ≥ 0.05.

### Renal denervation procedure

The RDN procedures were performed using MarinR 5F (*n* = 14), Symplicity Flex (*n* = 23), Vessix Reduce (*n* = 7), and Symplicity Spyral (*n* = 15) catheters in accordance with the manufacturer’s instructions, except that the treatments were performed mainly in the distal branches of the renal artery and, in some cases, extended proximally to the distal trunk if one or more branches were unsuitable for the treatment. The average number of ablations on each side ranged from 4 to 12. The mean number of point treatments delivered per patient was 13.1 ± 1.66. The number of ablations depended on the diameter of the artery (4–if the branch had a diameter of 4 mm or greater and 2–if the diameter was less than 4 mm). If less than 4 treatments were performed in the branch, 2 additional treatments were done in the distal trunk to achieve sufficient completeness of the intervention. Two subjects had accessory renal arteries (diameter was less than 4 mm) that were successfully treated.

### Statistical analysis

Statistica 10.0 ver. and SPSS 26 for Windows were used for the statistical analysis. The significance of differences in categorical variables was tested using Fisher’s exact test. The Shapiro–Wilk test was used to test the hypothesis of a normal distribution of continuous variables. Between-group differences were tested using unpaired *t*-tests for normally distributed variables and the Mann–Whitney *U* test otherwise. Within-group differences in repeated measures were assessed using paired *t*-test or Wilcoxon signed-rank test. The association between variables was assessed using Pearson’s correlation coefficient (r). The 95% confidence intervals (CI) were calculated to assess the size of the treatment effects according to the International Conference on Harmonization E9 Guideline: “Statistical Principles for Clinical Trials”. Analyses were performed based on the per-protocol principle. Statistical significance was set at *p* < 0.05.

## Results

The clinical characteristics of the patients are summarized in Table 1. The procedure was performed without any complications in 96.6% of the patients. Two patients had complications (one femoral arterial pseudoaneurism and one subcutaneous hematoma), all of which resolved without extending the terms of hospitalization. No major safety concerns were observed.

**TABLE 1:** Clinical characteristic [M ± SD, n (%)].

Parameters	All patients (*n* = 59)	Group 1 RRI ≥ 0.7 (*n* = 23)	Group 2 RRI < 0.7 (*n* = 36)	p_*g1*–*g2*_
Age, years	60.3 ± 7.9	65.0 ± 6.2	57.4 ± 7.5	0.001
Sex, female	34 (57.6)	17 (73.9)	17 (47.2)	0.038
Body mass index, kg/m^2^	35.2 ± 6.0	34.3 ± 5.2	35.7 ± 6.4	0.404
Duration of hypertension, years	22.1 ± 9.8	24.6 ± 10.4	20.5 ± 9.2	0.127
Duration of diabetes mellitus, years	9.7 ± 6.5	10.5 ± 6.6	9.1 ± 6.5	0.434
Coronary artery disease	37 (62.7)	18 (78.3)	19 (52.8)	0.043
History of myocardial infarction	10 (17.0)	4 (17.4)	6 (16.7)	0.604
History of stroke	11 (18.6)	4 (17.4)	7 (19.4)	0.564
Isolated systolic HTN	24 (40.7)	16 (69.6)	8 (22.2)	0.001
Office SBP, mm Hg	170.7 ± 19.8	173.1 ± 19.9	169.3 ± 1.9	0.475
Office DBP, mm Hg	88.2 ± 14.5	80.2 ± 13.3	93.3 ± 13.0	0.001
Office HR, bpm	70,0 ± 9.2	68.3 ± 8.4	71.2 ± 9.6	0.246
24-h ambulatory SBP, mm Hg	158 ± 16.3	159.4 ± 15.3	157.1 ± 17.4	0.604
24-h ambulatory DBP, mm Hg	82.5 ± 12.7	76.3 ± 13.9	86.5 ± 10.2	0.002
24 h mean pulse pressure, mm Hg	76,0 ± 14,2	83.1 ± 11	71.4 ± 14.2	0.002
HbA1c, %	7.5 ± 1.5	7.7 ± 1.4	7.3 ± 1.6	0.349
FPG, mmol/L	8.8 ± 2.7	8.5 ± 2.7	8.9 ± 2.8	0.598
eGFR (CKD-EPI), ml/min/1.73 m^2^	69.7 ± 22.2	57.3 ± 19.6	77.6 ± 20.2	0.001
Renal resistive index	0.676 ± 0.080	0.756 ± 0.043	0.623 ± 0.052	0.001
Chronic kidney disease	30 (54.3)	16 (69.6)	14 (38.9)	0.020
No. of antihypertensive drugs	4.3 ± 1.0	4.5 ± 1.0	4.3 ± 1.0	0.396

Data are presented as number (%), or mean ± standard deviation. SBP, systolic blood pressure; DBP, diastolic blood pressure; HR, heart rate; HbA1c, glycated hemoglobin; FPG, fasting plasma glucose; eGFR, estimated glomerular filtration rate.

**FIGURE 2:**
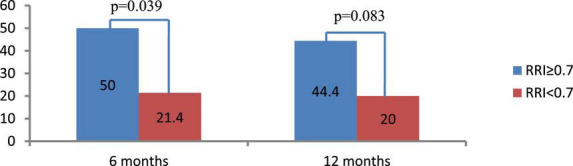
Frequency of renal resistive index (RRI) decrease by ≥ 0.05 at 6 and 12 months after renal denervation (RDN). Data are presented as number (%).

Forty-eight and forty-three patients completed the 6-and 12-months follow-up, respectively. The change in the RRI was strongly dependent on the baseline level. Specifically, the RRI significantly decreased in patients with elevated baseline levels of RRI ≥ 0.7 [−0.024 (95% CI: −0.046, −0.002), *p* = 0.011 at 6 months, and −0.024 (95% CI: −0.046, −0.002), *p* = 0.035 at 12 months] but only slightly changed in those with baseline RRI < 0.7 [0.009 (95% CI: −0.016, 0.035), *p* = 0.458 at 6 months and remained insignificant at 12 months after RDN 0.024 (95% CI: −0.002, 0.050), *p* = 0.069]. The frequency of improvement in renal vascular resistance (defined as a decrease in RRI by > 0.05) was almost two times higher in the first group at 6 and 12 months follow-up than in the second group (Figure 2).

Retrospectively, the two groups of patients differed in some baseline clinical characteristics (Table 1). The group with RRI > 0.7 included 23 patients (39%) who were mostly women, significantly older (8 years on average), had lower eGFR values (on average by 23.3 mL/min/1.73 m^2^), and a higher incidence of CKD (1.8 times). They also had higher pulse pressure levels and a higher frequency of ISHTN (3.6 times) and coronary artery disease (1.5 times). The number and spectrum of antihypertensive and antidiabetic therapies used in both groups were not significantly different (Table 2).

**TABLE 2:** Characteristics of antihypertensive and antidiabetic therapy.

Parameters	All patients (*n* = 59)	Group 1 RRI ≥ 0.7 (*n* = 23)	Group 2 RRI < 0.7 (*n* = 36)	p_*g1*–*g2*_
**Antihypertensive therapy**				
Beta-blockers	49 (83.1)	20 (87.0)	29 (80.6)	0.770
ACEI/ARBs	55 (93.2)	21 (91.3)	34 (94.4)	0.510
Diuretics	57 (96.6)	22 (95.7)	35 (97.2)	0.631
Calcium channel blockers	46 (78.0)	19 (82.6)	27 (75.0)	0.362
Spironolactone	37 (62.7)	14 (60.9)	23 (63.9)	0.515
I1-imidazoline receptor agonists	15 (25.4)	8 (34.8)	7 (19.4)	0.288
Alpha-blockers	8 (13.6)	3 (17.1)	5 (13.9)	0.700
**Antidiabetic therapy**				
Dietotherapy	5 (8.5)	1 (4.4)	4 (11.1)	0.346
Insulin	20 (33.9)	11 (55)	9 (25)	0.064
Metformin	15 (25.4)	4 (17.4)	11 (30.6)	0.206
Gliclazide	7 (11.9)	4 (17.4)	3 (8.3)	0.259
Combined oral antidiabetic therapy	12 (20.3)	3 (13.0)	9 (25.0)	0.219
Statins	58 (98.6)	23 (100)	35 (97.2)	0.610

Data are presented as number (%), or mean ± standard deviation. ACEI/ARBs, angiotensin-converting enzyme inhibitors/angiotensin receptor blockers.

**FIGURE 3:**
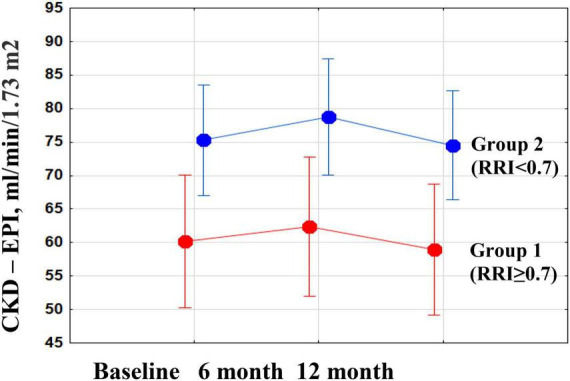
Changes in estimated glomerular filtration rate (eGFR) during the year after renal denervation (RDN) in groups of patients with high and normal renal resistive index (RRI).

**FIGURE 4:**
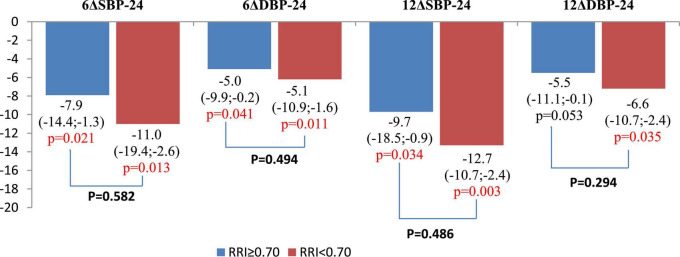
Comparison of changes in 24-h ambulatory blood pressure (BP) values at 6 and 12 months after renal denervation (RDN). Data are mean and 95% confidence interval.

There were no significant changes in eGFR, FPG, and HbA1c in either group during the year of follow-up, as well as in inter-group differences (Figure 3; Table 3). RDN reduced systolic and diastolic BP at 6 and 12 months by −10.3 (95% CI: −15.9, −4.7)/−10.9 (95% CI: −16.7, −5.0) and −5.8 (95% CI, −9.1 to −2.4)/−5.5 (95% CI: −8.7, −2.4) mmHg, respectively. Twenty-seven patients (62.7%) had a 24-h ambulatory systolic BP reduction of > 10 mmHg after 12 months (defined as a response). The number of drugs did not change (from 4.3 ± 1.0 to 4.3 ± 1.1, *p* = 0.811). The BP changes were significant and comparable in both groups during the follow-up period (Figure 4).

**TABLE 3:** Changes in estimated glomerular filtration rate (eGFR), fasting plasma glucose (FPG), and glycosylated hemoglobin (HbA1c) at 12 months after renal denervation (RDN).

	RRI ≥ 0.7	*p*	RRI < 0.7	*p*
eGFR (CKD-EPI), ml/min/1.73 m^2^	−2.7 [−3.0…8.4]	0.330	−0.8 [−5.0…3.5]	0.713
FPG, mmol/L	0.12 [−0.92…1.17]	0.804	−0.22 [−0.70…0.25]	0.337
HbA1c, %	0.11 [−0.41…0.64]	0.660	−0.13 [−0.80…0.53]	0.674

Data are mean and 95% confidence interval.

The decrease in RRI did not correlate with systolic BP reduction after RDN (*r* = 0.05, *p* = 0.73 at 6 months; and *r* = 0.08, *p* = 0.59 at 12 months).

According to multiple regression analysis (Table 4) level of Hb1Ac had an independent effect on the RRI response to RDN.

**TABLE 4:** Multivariate analysis of the factors influencing changes in renal resistive indexes from baseline to 6 months after renal denervation (RDN).

Variable	Estimate parameter	Standard error	*p*
age	0.0005	0.0012	0.6459
Baseline Hb1Ac	0.0243	0.0082	0.0051
Baseline 24-h ambulatory SBP	0.0006	0.0006	0.3349
Male	–0.0005	0.0185	0.9787
Baseline eGFR	0.0006	0.0004	0.1895

## Discussion

Our study is the first to analyze the effects of RDN on renal vascular resistance in patients with RHTN and T2DM. The positive effect of renin-angiotensin-aldosterone system (RAAS) blockade and sodium-glucose co-transporter 2 (SGLT2) inhibitors on renal hemodynamics in patients with HTN and T2DM has been previously described (22, 23). It should be noted that our patients did not use SGLT2 inhibitors, but all of them received RAAS inhibitors. Despite this, 39% of the patients had an increased RRI. A possible reason for the increase in renal vascular resistance may be sympathetic-mediated vasoconstriction, as earlier studies have shown that RRI depends on sympathetic tone (24–26).

Several studies have demonstrated sustained reduction in muscle sympathetic nerve activity (27, 28) and decreased noradrenaline spillover after RDN (29). Renal vascular tone is largely mediated by renal sympathetic nerve activity, and it may be expected that RDN-induced blockage of renal nerves can effectively decrease renal vascular resistance and RRI. Previously, Mahfoud et al. also showed a significant decrease in RRI at 3 and 6 months after RDN in the general population of hypertensive patients, independent of BP reduction (30). However, our study is the first to assess the effect of RDN on RRI in a selected group of patients with RHTN and T2DM.

The main finding of our study is that the effect of RDN on renal vascular resistance in patients with RHTN and T2DM strongly depends on its baseline status, with a pronounced decrease in RRI in patients with elevated baseline values ≥ 0.7 and no effect otherwise. It is also noteworthy that some patients showed regression in RRI without significant BP reduction. The possible mechanisms of the positive effect of RDN on renal vascular resistance may include not only a decrease in sympathetic tone but also suppression of chronic low-intensity inflammation (28, 29, 31, 32), which leads to dilation of the renal arteries.

This study has important clinical implications. Recently, Delsart et al. reported that an increase in RRI > 0.70 in hypertensive patients with T2DM and HTN over a 10-year follow-up remained a strong and independent predictor of cardiovascular and renal complications (5). Taking this and other evidences of the relationship between elevated RRI, target organ damage, and poor cardiovascular prognosis into account (5–10, 12, 13), RDN can be expected to provide additional benefits in patients with elevated renal vascular resistance independent of the BP-lowering effect.

Another important finding of our study is that RDN was accompanied by stabilization of eGFR in patients with RHTN and T2DM, which normally show a steady decline in this index by 2–4 mL/min/1.73 m^2^ annually (31). Concerning glucose metabolism, RDN had no impact on fasting glucose and HbA1C levels. Our data are consistent with the recent meta-analysis investigating the effects of catheter-based RDN on glucose metabolism (32).

In our study, we also described the clinical phenotypes of patients with high RRI. The high RRI phenotype was associated with older age and a high incidence of not only CKD and coronary artery disease but also ISHTN, which could reduce the BP response to RDN. However, according to our data, there was a significant decrease in BP after RDN in both groups, and the degree of BP response was comparable. Currently, information regarding the antihypertensive efficacy of RDN in patients with ISHTN is contradictory. In some studies, older age and ISHTN were predictors of BP nonresponse to RDN (33, 34). It has been suggested that a less pronounced response to RDN in patients with ISHTN may be associated with an increase in vascular stiffness and a lower possibility of reverse vascular remodeling. At the same time, according to the results of the Global SYMPLICITY Registry, the presence of ISHTN did not affect the degree of BP reduction (18). It can be assumed that an increase in renal vascular resistance, as well as the presence of CKD in patients with ISHTN, is associated with activation of the sympathetic nervous system, which determines the BP response to RDN.

### Limitations

There were several limitations in this study. First, this was a single-arm study with no sham-control group. However, several rigorously controlled studies of RDN have previously shown a quite insignificant effect of the sham procedure on BP at 3–6 months after the intervention (35–37). Second, the small sample size may not have provided enough power to detect some between-group differences in secondary outcomes, as well as additional predictors of RRI response to RDN. Third, our sample only included the patients with combination of RHTN and T2DM, therefore, it was impossible to assess if and how the detected response of RRI to RDN depends on the presence of T2DM. Future studies are needed to address this important question. Finally, the results of this exploratory study should be further evaluated in larger randomized control trials.

## Conclusion

In summary, we showed that RDN can decrease elevated RRI 0.7 and stabilize kidney function in patients with RHTN and T2DM independently of its BP-lowering effect. Long-term follow-up is needed to assess cardiovascular and renal outcomes depending on changes in RRI after RDN in patients with RHTN and T2DM. Further research may be needed to study the relationship between changes in RRI and changes in markers of sympathetic activity and chronic low-degree inflammation. Considering that SGLT2 inhibitors reduce sympathetic nervous system activity (38), future randomized trials are needed to compare changes in renal hemodynamics after RDN versus a combination of RDN and SGLT2 inhibitors.

## Data availability statement

The original contributions presented in this study are included in the article/supplementary material, further inquiries can be directed to the corresponding author.

## Ethics statement

The studies involving human participants were reviewed and approved by Biomedical Ethics Committee of Cardiology Research Institute, Tomsk NRMC. The patients/participants provided their written informed consent to participate in this study.

## Author contributions

MM, AF, VM, and StP contributed to conceptualization, participated in the development of a general concept and research design, obtained, analyzed, and interpreted the data, and wrote the first version of the manuscript and prepared it for publication. AF, SeP, and VM contributed to methodology. MM and AF contributed to software. ES, TR, SK, and AV formed a sample of patients, organized data collection, and contributed to the revision of the original manuscript. All authors contributed to the article and approved the submitted version.
